# Supplementary material for: Conditional chemoconnectomics (cCCTomics) as a strategy for efficient and conditional targeting of chemical transmission
Source: eLife. 2024 Apr 30;12:RP91927. doi: 10.7554/eLife.91927 (PMC11060718; doi:10.7554/eLife.91927)
Supplement: Figure 3—source data 3. [file elife-91927-fig3-data3.zip › Figure 3 source data3 part 1.zip!/Figure 3-Source data 3-part1/1067-9-CRISPResso_Report_c33kf3u3.zip!/CRISPResso_on_1067_10/1c.Alignment_barplot.pdf]

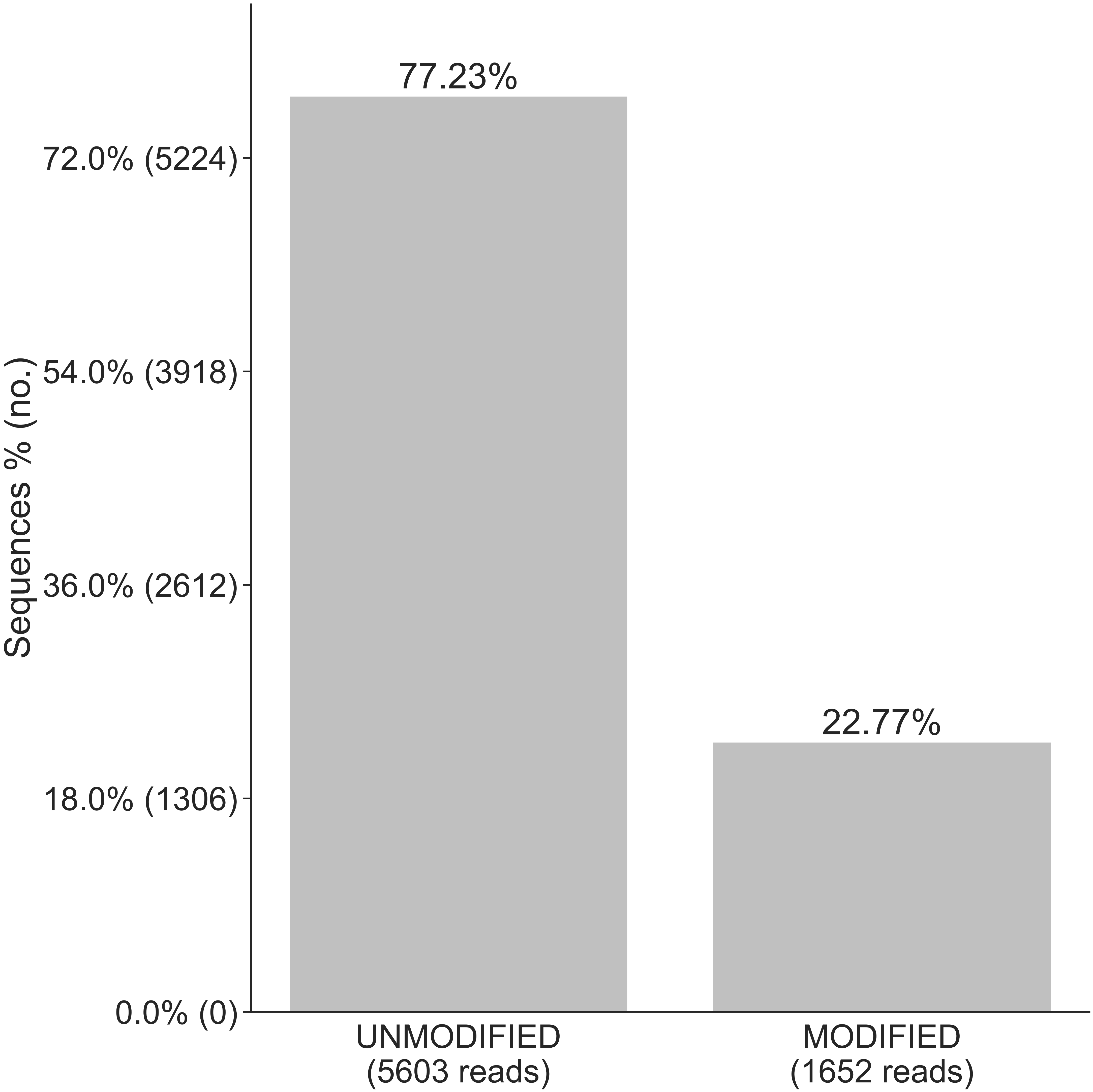
77.23%
22.77%
72.0% (5224)
54.0% (3918)
36.0% (2612)
18.0% (1306)
0.0% (0)
Sequences % (no.)
UNMODIFIED
(5603 reads)
MODIFIED
(1652 reads)
